# Sustainability of Enzymatic Monomer Synthesis: Evaluation via Comparison of Petrochemical and Enzymatic Alkene Epoxidation by Life Cycle Assessment

**DOI:** 10.1002/cssc.202402248

**Published:** 2025-02-04

**Authors:** Robin Tannert, Sarah Barth, Jakob Hildebrandt, Andreas Taubert, Jens Weber

**Affiliations:** ^1^ ZIRKON Hochschule Zittau/Görlitz (University of Applied Sciences) Theodor-Körner-Allee 16 02763 Zittau Germany; ^2^ Institute of Chemistry Universität Potsdam Karl-Liebknecht-Straße 24–25 14476 Potsdam Germany; ^3^ Faculty of Natural and Environmental Science Hochschule Zittau/Görlitz (University of Applied Sciences) Theodor-Körner-Allee 16 02763 Zittau Germany

**Keywords:** Sustainable chemistry, Catalysis, CAL-B, Epoxidation, Life cycle assessment

## Abstract

Life cycle assessment (LCA) was used, next to green chemistry concepts, to compare the full environmental impacts of the epoxidation of a bio‐based monomer, which can be used for the synthesis of vitrimers. On a laboratory scale, the synthesis of the monomer can either be done via a petrochemical route or via an enzymatic reaction pathway. Both reaction pathways were initially optimized to minimize the impact of suboptimal routes on the sustainability evaluation. The subsequent assessment of the enzymatic routes shows lower impact factors for most criteria compared to the petrochemical routes. A significant drawback of the enzymatic reaction, however, is its electricity consumption. The yields of the respective reactions also proved to be crucial; realistic changes in yields revealed the petrochemical reaction to be more sustainable in some cases. LCA is therefore a valuable tool for the preliminary evaluation of the developed synthesis pathways and to identify the critical adjustments needed to increase the sustainability of each reaction.

## Graphical abstract

How sustainable are enzymatically catalyzed reactions really? This study performs a life‐cycle assessment (LCA) using an enzymatically catalyzed epoxidation as an example and compares the results with a petrochemical counterpart of the identical molecule. We demonstrate that LCA is an essential tool for more accurately evaluating the sustainability of reactions.

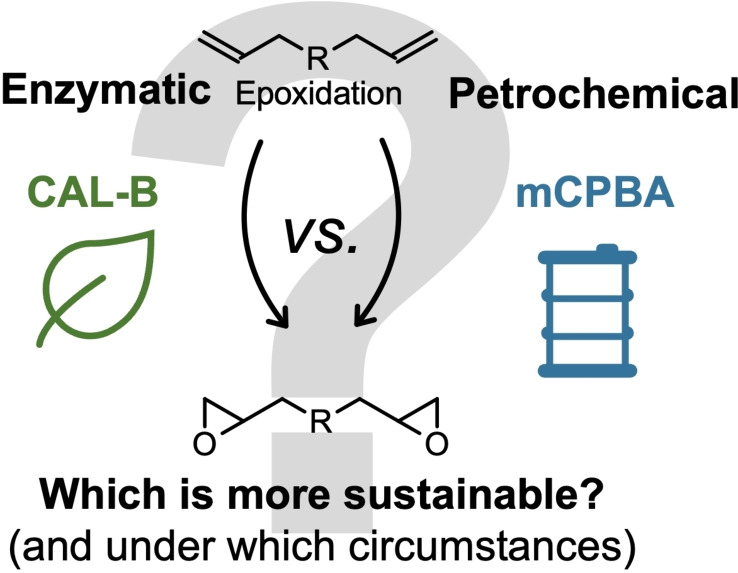

## Introduction

Epoxides are widely used in chemistry. They are well known reactive intermediates in synthetic organic chemistry as well as monomers in polymer chemistry.[^1^, ^2^, ^3^, ^4^, ^5^, ^6^, ^7^, ^8^] Epoxides can be synthesized via two main pathways: the etherification of alcohols with epichlorohydrin (commonly performed with bisphenol A to obtain diglycidyl ethers as monomers in polymer chemistry) and the oxidation of alkenes via a *Prilezhaev* reaction.[^9^, ^10^] In the *Prilezhaev* reaction oxygen is transferred from a peroxy acid in a single step to the alkene yielding the epoxide.[^10^, ^11^, ^12^] This reaction is widely used, often utilizing 3‐chloroperbenzoic acid (meta‐chloroperbenzoic acid, mCPBA) due to its safe handling and high epoxidation potential.[^2^, ^4^, ^6^, ^7^, ^13^, ^14^] However, because of the equivalent consumption of the peroxy acid, stoichiometric amounts are needed. Moreover, mCPBA is an aromatic and chlorinated chemical, thus raising serious concerns regarding its sustainability and toxicity. Following the concepts of *green chemistry*, this reaction clearly shows a need for improvement.^[15]^

Alternative pathways for epoxidation exist, notably the use of various metal catalysts combined with hydrogen peroxide.[^16^, ^17^] Furthermore, enzymes such as *Candida antarctica lipase B* (CAL‐B) can also be used for epoxidation of alkenes.[^18^, ^19^, ^20^, ^21^, ^22^, ^23^] In this process, the enzyme does not directly epoxidize the alkene but requires a co‐substrate – typically an aliphatic organic acid and hydrogen peroxide – to form a peroxy organic acid that subsequently epoxidizes the alkene. The peroxy acid is formed by the reaction of hydrogen peroxide with the acyl‐enzyme‐complex, which consists of the aliphatic acid and the hydroxy group of the amino acid serine in the active site.[^21^, ^24^, ^25^] The *in situ* formation of the peroxy acid allows for a safer handling and is used for the epoxidation of alkenes as well as for the *Baeyer‐Villiger* oxidation.[^18^, ^19^, ^20^, ^21^, ^22^, ^23^, ^25^, ^26^, ^27^, ^28^]

Using enzymatic reactions satisfies several concepts of *green chemistry*:^[15]^ (1) avoiding mCPBA results in a less hazardous synthesis, replacing it with an aliphatic acid, hydrogen peroxide, and an enzyme in the enzymatic reaction; (2) renewable materials (enzyme, hydrogen peroxide) are used, which thereby avoid a depleting feedstock; (3) a catalyzed reaction is applied, which is generally viewed superior to stoichiometric reagents.

However, the simple application of these rules alone does not define a reaction as truly sustainable, as environmental impacts are more complex. The impact of the production of the chemicals needs to be considered as well as the energy for the enzymatic reaction, as these are key factors for a (more) complete evaluation of the sustainability of a reaction. Furthermore, a bunch of additional criteria accumulate to a total environmental impact, which might not all be reduced during a common process optimization that does only look at improved yield or selectivity. Changes in a reaction procedure (including work‐up) might affect a chosen criterion in a positive way, while others might be affected negatively at the same moment.^[29]^

An established way to evaluate the sustainability of a certain process or reaction is to conduct a life cycle assessment (LCA). This assessment delivers a more comprehensive result as it considers the impacts of the utilized chemicals for the reaction as well as the necessary energy input and provides a result reflecting impacts and climate effects, material use and also environmental toxicity.^[30]^

**Figure 1:**
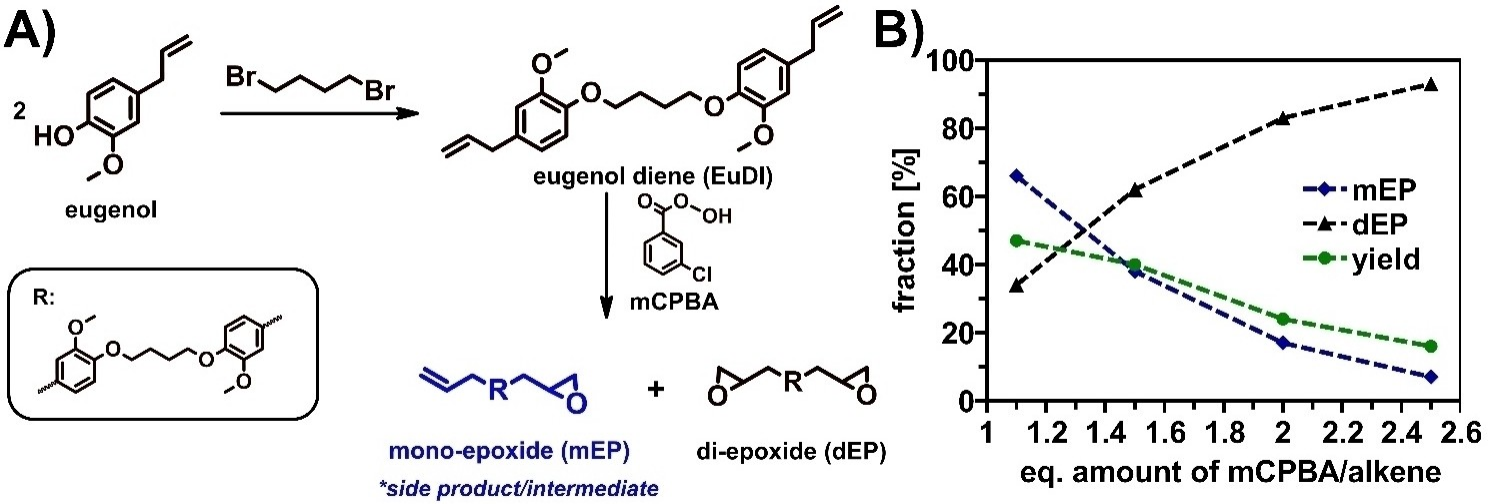
A) synthesis of diepoxidized eugenol derivative by dimerization of eugenol via etherification with 1,4‐dibromobutane followed by epoxidization with mCPBA, yielding monoepoxidized derivative (mEP) (intermediat) and diepoxidizied derivate as final product, B) fraction of mEP and dEP and yield (mEP+dEP) depending on the stoichiometric excess of mCPBA.

Previously, we have reported the synthesis of bio‐based vitrimers using a diepoxide monomer prepared by epoxidation from an eugenol‐based molecule using mCPBA (see Figure 1).^[31]^ During the course of the monomer synthesis, we found that yields of the diepoxide species were rather low. Low yields prompt the question if a bio‐based monomer is still sustainable, if over‐stoichiometric amounts of an aromatic, chlorinated species are needed for its synthesis. Hence, we conducted the alkene (terminal CC double bond) epoxidation of the eugenol‐based species now in two ways, one via the common way by the use of mCPBA and the other via the use of the enzymatically catalyzed reaction with CAL‐B.^[14]^ Both reactions were optimized regarding their efficiency, minimal resource utilization and a high yield. At the end of the process, we obtained two optimized procedures (on a laboratory scale) yielding the same product but via two different reaction pathways: chemical epoxidation with mCPBA *vs*. enzymatic epoxidation. Thus, the two reactions are ideal for a comprehensive comparison of their sustainability by LCA. Furthermore, as enzymatic reactions are generally assumed to be more sustainable (following the rules of *green chemistry*), the current data allow for a detailed discussion whether this rather general assumption is correct in this specific case.

## Results and Discussion

### Petrochemical Reaction: Epoxidation with mCPBA

The target molecule for this reaction method optimization and LCA is an epoxidized eugenol‐derivative (dEP), originally synthesized by Liu *et al*, shown in Figure 1A.^[14]^ However, we found that the epoxidation reaction does not run smoothly, as a significant amount of a side product – the intermediate monoepoxidizied derivative (mEP) – was present in the resulting product following the reported procedure.^[31]^

Due to severe difficulties in separating these two, chemically quite similar substances, we adapted the reaction conditions. Increasing the amount of mCPBA to overstoichiometric amounts of 2.5 mCPBA molecules per terminal C=C double bond improved the epoxidation degree significantly to >90 %. However, with this adaptation, the overall yield dropped strongly, resulting in only approximately 20 % of dEP (see Figure 1B).

Further optimization of the reaction conditions was undertaken to either improve the conversion or the yield. A change in temperature from room temperature to 0 °C due to stability concerns of mCPBA revealed no improvement, only a decline in the degree of conversion to traces of the designated epoxide dEP. Other optimizations, including changing the atmosphere from air to nitrogen, stepwise addition of mCPBA over an extended period (three additions, one per day), or increasing the reaction time (up to one week) also showed no improvements. Further analyses showed that a crucial side reaction occurred during the process, which accounted for the high consumption of mCPBA and the low yield.

### Biotechnological Reaction: Enzymatic Epoxidation

**Scheme 1:**
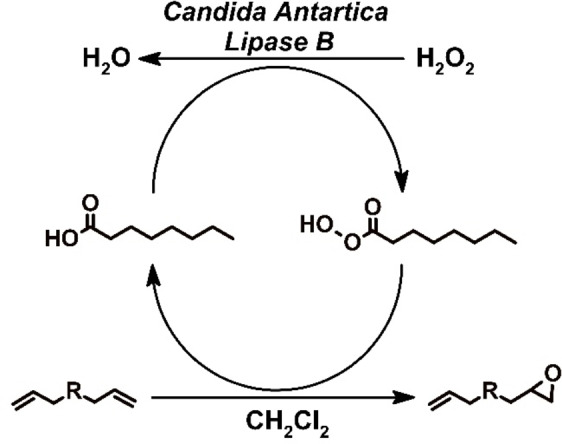
catalytic cycle for the C=C double bond epoxidation via the enzymatic oxidation of the cosubstrate octanoic acid by CAL‐B using hydrogen peroxide.

An alternative reaction pathway to epoxidize the terminal C=C double bond is the utilization of the *Candida antarctica Lipase B* (CAL‐B), which oxidizes a co‐substrate (e. g. octanoic acid) under the consumption of hydrogen peroxide (Scheme 1).[^19^, ^20^, ^21^, ^24^, ^26^, ^32^, ^33^] Some enzymes catalyze the oxidation of acids to the corresponding peroxy acids; among them, CAL‐B shows the best performance.[^19^, ^21^, ^26^] There are numerous protocols available for epoxidations using CAL‐B, however they differ strongly in the reaction details.[^18^, ^19^, ^20^, ^22^, ^25^] As the enzymatic epoxidation of EuDI was not reported before, we decided to run an optimization of the synthesis regarding temperature, concentrations and reaction time.

We began by adapting a method from *Schneider et al*., who reported the epoxidation of sunflower oil methyl esters in a biphasic system of dichloromethane and water.^[20]^ As EuDI has sufficient solubility at room temperature unfortunately only in chlorinated solvents such as chloroform or dichloromethane, this method was the closest choice. Applying the reported reaction conditions to our system resulted in 35 % conversion of all terminal C=C double bonds to epoxides (reaction mixtures consisting of 50 % mEP and 10 % dEP). Repeating the reaction under the same conditions but excluding the enzyme as a control reaction resulted in no epoxide formation, clearly demonstrating the enzyme′s catalytic effect.

### Temperature

**Figure 2:**
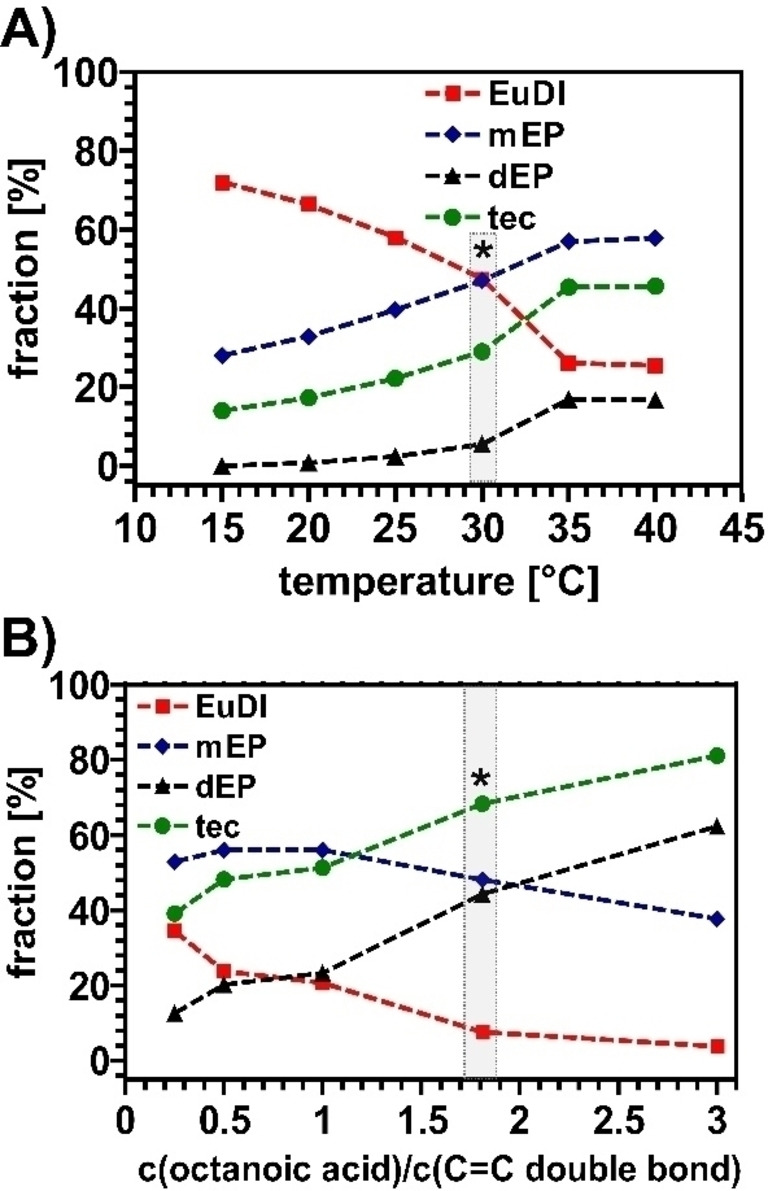
fraction of EuDI (red), mEP (blue), dEP (black) and total epoxide conversion (tec, green) under different reaction conditions after EuDI epoxidation with CAL‐B, A) optimization of temperature: C_EuDI_=50 g/L, C_CAL‐B_=2.5 g/L, CO_octanoic acid_=1.8 mol/C=C, C_H2O2_=2 mol/C=C, 20 hours, solvent: 1 : 1 mixture dichloromethane:water, B) optimization of co‐substrate concentration: C_EuDI_=50 g/L, C_CAL‐B_=2.5 g/L, C_H2O2_=2 mol/C=C, 20 hours, θ=35 °C, solvent: dichloromethane; *marks the starting point of the respective optimization series.

First, we investigated the influence of the reaction temperature from 15 °C to 40 °C while keeping the other reaction parameters constant. The influence of the reaction temperature is shown in Figure 2A.

With increasing temperature, a steady increase in enzyme activity (judged by the total epoxide conversion (tec), which represents the fraction of initial C=C double bonds converted into epoxides) could be observed up to 35 °C; thereafter, no further improvement could be observed. Enzyme deactivation and fast hydrogen peroxide decomposition with higher temperature should not be the reason for the absence of a further increase, as other enzymatic epoxidation with the same principle were reported using higher temperatures.[^18^, ^22^, ^34^] Furthermore, a general increase in enzyme activity with steadily rising temperature cannot be expected, as lower temperatures have also been reported as the optimum for CAL‐B enzyme activity, depending on the system.^[19]^

### Solvents

The variation of the solvent was strongly limited because of the restricted solubility of EuDI, which is only soluble in dichloromethane and chloroform. The solvent system also contained water, based on the observations from *Schneider et al* who demonstrated that usind a biphasic solvent system helps preserve enzyme activity and enables potential reusability.^[20]^ Precipitation of some solid could be observed after some time because of the changed polarity due to the biphasic system, which was inconvenient for us. Since there was no positive effect on the enzyme reusability due to the biphasic system (regardless of the solvent system, tec dropped below 5 % with recycled enzyme), we excluded any additional water (with exception from the water added with the addition of hydrogen peroxide) from the reaction, which increased the degree of conversion from 45 % (58 % of mEP and 17 % of dEP, see Figure 2A, T=35 °C) to 68 % (48 % for mEP and 44 % for dEP, see Figure 2B, c_octanoic acid_=1.8 mol/C=C double bond). The results indicate a negative effect of additional water on the reaction progress.

### Co‐Substrate

Variation of the type and concentration of different co‐substrates (normally aliphatic organic acids) might also affect the enzymatic catalysis. Numerous reports have already shown that octanoic acid is the most suitable co‐substrate type and is usually showing the best results.[^19^, ^35^, ^36^] So, we decided to directly use octanoic acid as co‐substrate. The influence of different octanoic acid concentrations on the total epoxide conversion is shown in Figure 2B. A clear increase in total epoxide conversion can be seen at higher concentrations of octanoic acid. The trend suggests that a further increase of the octanoic acid concentration might yield even higher conversion, we decided to stop at the threefold stoichiometric excess of octanoic acid compared to the terminal C=C double bonds to prevent higher amounts of waste. Other studies showed that the co‐substrate indeed does not act as a co‐catalyst and must be used in higher amounts (around equivalent substrate concentrations) to achieve a sufficient conversion.[^18^, ^19^]

### Hydrogen Peroxide

**Figure 3:**
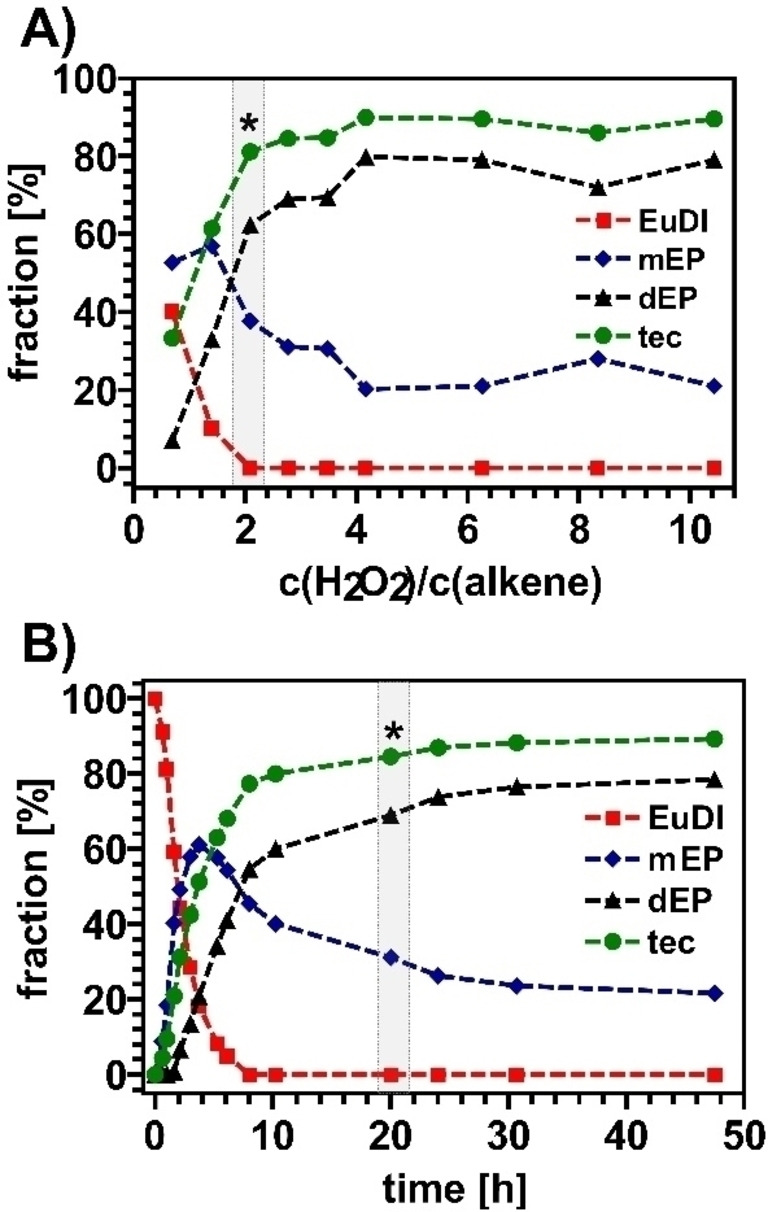
fraction of EuDI (red), mEP (blue), dEP (black) and total epoxide conversion (tec, green) under different reaction conditions after EuDI epoxidation with CAL‐B, A) optimization of hydrogen peroxide concentration: C_EuDI_=50 g/L, C_CAL‐B_=2.5 g/L, C_octanoic acid_=3 mol/aC=C, 20 hours, θ=35 °C, solvent: dichloromethane, B) optimization of reaction time: C_EuDI_=50 g/L, C_CAL‐B_ =2.5 g/L, C_octanoic acid_=3 mol/C=C, C_H2O2_=4 mol/C=C, θ=35 °C, solvent: dichloromethane; *marks the starting point of the respective optimization series.

Another optimization parameter is the concentration of hydrogen peroxide (see Figure 3A). As we already observed that the presence of additional water did hinder the reaction, we used lower concentration of hydrogen peroxide to improve the epoxide conversion. However, too low concentrations (equimolar) resulted in only low conversions. To provide a sufficient concentration of H_2_O_2_ in the organic phase, excess concentrations of at least two molecules of H_2_O_2_ per terminal C=C double bond were necessary.^[22]^ Slight increases for the degree of conversion could be observed for even higher hydrogen peroxide concentration, showing an upper limit at around a fourfold stoichiometric excess. Even higher concentrations did neither result higher conversion, nor did they inhibit the reaction.

The time of the addition of hydrogen peroxide in portions was also varied to check for or compensate for the degradation of hydrogen peroxide because of its limited stability. We conducted the reaction with a fourfold stoichiometric excess of H_2_O_2_ (in total), where half of the total amount was added in two equal portions after 24 and 48 h, respectively. However, no further improvement of the conversion could be observed.

### Reaction Time

The final parameter studied for optimization was the reaction time. The kinetics of the reaction are shown in Figure 3B. The analysis of the kinetics revealed that the initially chosen time of 20 h reaction time was already almost sufficient. While the first 10 h showed a clear and straightforward progress, only slight increases could be observed after passing the 20 h mark. No further changes were observed after 30 h reaction time, making this the ideal reaction time.  The analysis of the reaction mixture composition clearly showed that the educt was entirely consumed. The total epoxide conversion was 91 %, due to a still non complete epoxidation of all double bonds (20 % of the monoepoxidized species of mEP are present in the mixture). The addition of additional hydrogen peroxide did not change this fraction, meaning that the incomplete epoxide conversion was not caused by a shortage of hydrogen peroxide (because of consumption and potential degradation) but most probably due to the inactivation of the enzyme CAL‐B itself.

### Further Reaction Adaption and Initial Sustainability Evaluation

Having the intention to compare the two different reaction pathways with each other, both need to be optimized regarding their *overall* procedure, i. e. workup of the mixture. This includes aspects like yield and chemicals consumption as well as the required energy for the total reaction procedure.

The epoxidation with the use of mCPBA, further referred to as the petrochemical reaction (PeR), was at an almost final optimized/adapted status (lab scale) where no further increase in yield could be achieved. As also the amount of solvents and other chemicals needed for work‐up contribute to the final assessment, the minimal amount of those chemicals that is needed to ensure that the reaction runs smoothly, was determined (Figure S1).

The optimization of the reaction conditions for the epoxidation using CAL‐B, further referred to as the enzymatic reaction (EnzR), was described above. The optimized conditions represent the ones resulting in the highest degree of conversion. However, we noticed that those were not beneficial for the final product isolation. After work‐up, the mixture contained a significant amount of octanoic acid, which could not be evaporated easily because of its elevated boiling point. For product isolation, a recrystallization was required to obtain the product in 24 % yield. Experiments were carried out with a reduced amount of octanoic acid to check for a potential increase in yield, as we suspected that the octanoic acid disturbed the crystallization process of the products (mEP and dEP). We noticed that the reduction of the threefold stoichoimetric excess to a twofold excess led to a crystallization of the product already during evaporation of the solvent dichloromethane. Although this leads to somewhat lower total epoxide conversion (see Figure 2B), it can be regarded as very beneficial in three points in terms of overall sustainability: (1) the product could be simply collected by filtration thus avoiding additional energy for recrystallization, (2) an increase in isolated yield to 60 % and (3) a lower amount of co‐substrate is needed, resulting in less waste.

**Figure 4:**
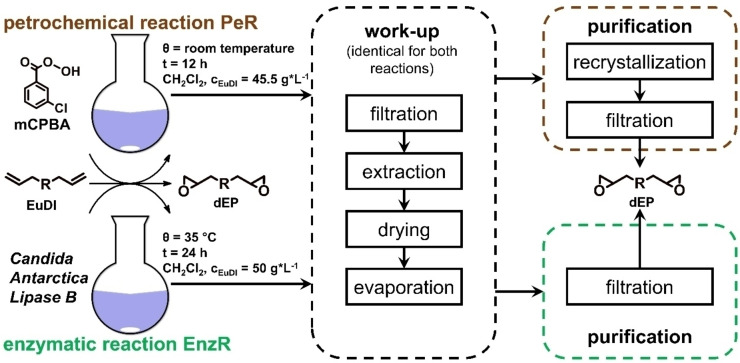
simplified scheme for reaction procedure for both PeR and EnzR, divided into three phases by reaction, work‐up (similar for both reactions) and purification.

The overall reaction procedure for both reactions is shown in Figure 4, detailed information are given in Figure S1 and Figure S2 (supporting information). These show all included chemicals with precise information of the used amounts and times. Included therein are also material flows, which are later implemented in the models for LCA.

A summary of main reaction parameters including key processing conditions as well as first parameters to compare and evaluate the sustainability of the reaction is given in Table 1. These allow for a first estimation of the sustainability for both reactions. According to the rules of *green chemistry*, the enzymatic reaction has to be seen as the more sustainable reaction.^[15]^ Here, rule number three (less hazardous chemical synthesis) is fulfilled, as the chlorinated chemical mCPBA is substituted with CAL‐B, hydrogen peroxide and octanoic acid. Furthermore, rule number seven (use of renewable feedstocks) as well as rule number nine (catalysis) are fulfilled by the use of the biological catalyst CAL‐B. Also, rule number two (atom economy) is achieved, as the calculation for both reactions revealed a higher atom economy for the EnzR. Another concept for evaluation is the *E* factor, which balances the ratio between waste and the product mass of the reaction.^[37]^ Here, again the EnzR showed a lower and therefore better value, however, the value is still quite high overall. The values of the *E* factors of 63.77 (EnzR) and 120.97 (PeR) were in a typical range for pharmaceuticals, thus representing reactions with enormous waste.^[36]^ This is mainly due to the use of solvents, which, in the current design, are considered as waste and are not recycled. We therefore also calculated the *E* factor for a situation excluding the solvents to evaluate the impact of the reactants and co‐reactants only. Consequently, much lower E factors were obtained of 15.44 for PeR and 5.08 for EnzR, respectively, which are now in the typical range for fine chemicals. Again, the EnzR showed the lower value and thus presented the more sustainable reaction.

**Table 1:** reaction parameter and sustainability comparison factors of atom economy and E factor of the petrochemical epoxidation (PeR) and the enzymatic epoxidation (EnzR).

	Reaction time [h]	Reaction temperature [°C]	Yield (per 10 g EuDI) [g]	Power [kWh]	Atom economy^[a]^	E factor^[a]^	
PeR	12	21	2.42	0.7922	56.6	120.97^[b]^	15.44^[c]^
EnzR	24	35	5.98	2.6817	73.4	63.77^[b]^	5.08^[c]^

[a] calculations see supporting information, [b]solvents included, [c] solvents excluded.

While these rules and parameters imply a higher sustainability for the EnzR, an absolute assessment based solely on these parameters might be viewed as questionable. The reason for this is the disregard of the energy required for the reaction, as well as the impact of the production of the chemicals that are needed for the reactions themselves. To obtain a comprehensive understanding of the true sustainability of both reaction pathways – specifically by how much certain environmental impacts can be reduced or increased, both reactions should be assessed through a Life Cycle Assessment (LCA), which can address these aforementioned shortcomings.^[30]^

## Life‐Cycle Assessment

### Methodology

The present LCA conforms to the existing ISO standard (14044) and follows the typical LCA framework.

### Goal, Scope, and Functional Unit

The LCA shall directly compare the two different reaction pathways using either a petrochemical or an enzymatical reaction on a laboratory scale. The starting compound and the product are identical for both reactions. The main differences are the reaction conditions, including the chemicals used, reaction temperature, and reaction time. The work‐up of the reaction to obtain the designated product is included in the assessment. For both reactions, the work‐up is identical, while differences existed in the purification procedure (Figure 4). The functional unit was selected according to the product declaration rules of the Environmental Product Declarations (EPD), which suggest using a physical reference, such as a piece (part), mass (kg), length (m), area (m^2^), volume (m^3^). A functional unit of 10 g of the final product dEP was chosen.

### System Description and Boundaries

The boundaries of the assessment correspond to a cradle‐to‐gate system, as further applications of the epoxides are identical and the exact use case is unknown. The use phase and the end‐of‐life phases are not considered here. The working schemes, used as references (backbone) for developing the LCA methodology, are shown in Figure S1 and Figure S2. Material and chemical flows are depicted according to these working schemes. Outputs were not reused; all outputs were either transferred to the next process or, if not intended, considered as waste. A scenario analysis was carried out to evaluate the recycling of the outputs.

### Data Collection

During the accounting process, primary data was preferred.

1. Primary Data: The primary data was collected from experiments at the laboratory scale. The data was recorded directly on site. This applies to both the inputs and outputs of the chemical products and the energy consumption data. For energy consumption data, the electricity consumption of the laboratory equipment was measured (see Table S1, supporting information).
2. Secondary Data: The background data used for basic chemicals such as solvents, acids, etc., comes from the Ecoinvent 3.7.1 database. German or European data was preferred. The datasets used were expanded with the background data of the process during the initial input. For chemicals not available in the database, they were replaced with alternative chemicals or generic datasets. This includes the following chemicals: mCPBA – replaced by benzoic acid extended with 1 eq. chlorine, CAL‐B enzyme – replaced by enzyme production from potato starch.

### Data Quality

The data used is largely based on specific data or quality‐assured generic information. It is assumed that these are valid for the geographical region and the technology used. The Ecoinvent data sets also have to pass through a separate quality assurance process with an assessment of the variances. Characterization factors were used in umberto LCA+ in accordance with the selected LCIA EF 3.0 methodology. The plausibility check of the data in the energy and material flow model was carried out within the umberto model. The result is an average value of very good data quality for the primary data and good to medium data quality for the generic data sets. The slightly poorer assessment of the data quality for the generic data sets is due to the fact that in some cases the exact data was not available and alternative inputs had to be used. No data was used whose data quality was rated as poor or very poor.

### Methods and Environmental Impacts Assessed

The European Commission proposed the Product Environmental Footprint (PEF) and Organisation Environmental Footprint (OEF) methods as a common way of measuring environmental performance (EU Commission Recommendation 2021/2279).^[38]^ The PEF and OEF are the EU recommended Life Cycle Assessment (LCA) based methods to quantify the environmental impacts of products (goods or services) and organisations.

In accordance with the EF 3.0 methodology, important characterization factors were used to ensure the comparability of the LCIA results:

– Climate change (total), mapped via the GWP (Global Warming Potential) indicator
– Stratospheric ozone depletion, mapped via the Ozone Depletion Potential (ODP) indicator
– Acidification, mapped via the Acidification Potential (AP) indicator
– Eutrophication (freshwater, saltwater, land), mapped using the indicators Freshwater Eutrophication Potential (FEP), Marine Eutrophication Potential (MEP) and Terrestrial Eutrophication Potential (TEP)
– Photochemical oxidant formation, mapped via the indicator Photochemical Ozone Formation Potential (POFP)
– Depletion of abiotic resources, mapped via the indicators Depletion of Abiotic Resources (ADP) and (Environmental Footprint
– Resource, fossils, EF‐Res)
– Water use, mapped using the Water Deprivation Potential (WDP) indicator

### LCA Results

The potential impacts of both reactions relative to the functional unit of 10 g of product (dEP) were compared across three main categories: environmental impacts, health/toxicity, and resources. The results are presented in Table 2. We firstly provide a direct comparison of the LCA results for both reactions. This is followed by a more detailed analysis of the factors that influences the upstream processes and production, the impact of different phases of the overall reaction procedure, and the effects of the chemicals used. This detailed analysis aims at identifying the main negative contributors to the total criteria values. The presented values are for a laboratory scale, which is why they display significantly higher values than LCAs performed for industrial‐scale reactions/processes. However, comparisons with other LCAs conducted at a laboratory scale show values in a comparable order of magnitude.^[39]^ Therefore, the obtained results could be considered to be realistic.

**Table 2:** results of different criteria of LCA of petrochemical reaction (PeR) and enzymatic reaction (EnzR), divided into three categories: environmental impacts, health/toxicity and resources, higher values (and therewith less sustainable criteria) are highlighted in bold, values are normalized to 10 g dEP.

		PeR	EnzR
Environmental impacts			
Climate change [kg CO_2_‐eq]		**12.14**	6.82
	Fossil	**12.13**	6.61
	Land use	0.0058	**0.17**
	Biogenic	0.013	**0.035**

Eutrophication			
	Freshwater [kg PO_4_^3−^‐eq]	0.0033	**0.00379**
	Marine [kg N‐eq]	**0.0178**	0.009
	Terrestrial [mol N‐eq]	**0.177**	0.084
Acidification [mol H^+^‐Eq]		**0.093**	0.038
Ozone depletion [kg CFC‐11‐Eq]		1.2*10^−4^	5.5*10^−5^

Health/toxicity			
Ecotoxicity freshwater [CTUe]^[a]^		**334.6**	104.2
	Metals	**85.05**	33.54
	Inorganics	**195.43**	68.15
	Organics	**54.08**	2.52
Human toxicity carcinogenic [CTUh]^[a]^		4.94*10^−9^	1.84*10^−9^
	Metals	3.41*10^−9^	1.6*10^−9^
	Inorganics	8.77*10^−18^	1.04*10^−18^
	Organics	1.53*10^−9^	2.46*10^−10^
Human toxicity non‐carcinogenic [CTUh]^[a]^		3.53*10^−7^	1.13*10^−7^
	Metals	1.99*10^−7^	9.62*10^−8^
	Inorganics	1.5*10^−7^	1.63*10^−8^
	Organics	6.81*10^−8^	1.75*10^−9^
Ionising radiation [kBq U235‐Eq]		**0.512**	0.479
Particulate matter formation [disease incidence]		1.49*10^−6^	6.48*10^−7^
Photochemical ozone formation [kg NMVOC‐Eq]		**0.05**	0.0224

Resources			
Water [m^3^ world eq. deprived]		**3.22**	1.36
Energy resources non‐renewable [MJ. Net calorific value]		**149.7**	80.49
Material resources metals/minerals [kg Sb‐Eq]		8.67*10^−5^	3.16*10^−5^
Land use soil quality index		11.91	**15.75**

[a] CTU…comparative toxic unit, index e: environment, index h: human.

A general comparison of the data clearly shows that, after comprehensive assessment of both reactions, the EnzR generally exhibits lower values for most criteria, indicating it is the more sustainable reaction. This finding aligns with results found in other studies.[^39^, ^40^, ^41^] Therefore, this confirms the supposition derived from the application of the concept of green chemistry.

In the category of environmental impacts, a reduction of approximately 44 % of carbon dioxide emission (climate change) was determined. The assessment revealed that the main fraction of emissions originated from the use of fossil feedstocks. However, emissions from land use or of biogenic origin were slightly higher for EnzR but negligible compared to the total amount. The only exception in this category, where EnzR showed a higher value, was for freshwater eutrophication. Both values are due to the use of a generic data set for the production of an enzyme. These effects should be seen as critical, as the actual enzyme CAL‐B used was replaced by a generic data set for enzyme production from potato starch as this was the closest available data set within the database. The real values could therefore show a different result. For the other criteria, a reduction of up to half could mostly be determined for EnzR compared to PeR.

The category concerning impacts on health/toxicity uniformly showed higher values for PeR. For freshwater ecotoxicity, all criteria displayed significant values, with inorganics having the highest impact for both reactions. Regarding carcinogenic impacts on human toxicity, inorganics exhibited the least impact, while metals had the greatest impact. Non‐carcinogenic impacts on human toxicity were distributed similarly for PeR and showed an elevated value of metals for EnzR. Additionally, criteria such as ionising radiation, particulate matter formation and, photochemical ozone formation showed significantly higher values for the PeR. The toxicity impacts can be primarily attributed to the chlorinated chemicals. Since both reactions used dichloromethane as the main solvent, a corresponding impact on toxicity due to the solvent is expected. The reduction in solvent usage for the PeR is offset by the additional use of mCPBA, which contains chlorine and requires further chlorine for its production (mCPBA synthesis via oxidation of 3‐chlorobenzoyl chloride).

Regarding resource requirements for the reactions, most indicated a higher demand for PeR. The exception was found in the land use soil quality index, influenced by enzyme production. This impact has also to be seen as critical. Therewith, real values might show another outcome. For other values, a clear decrease of up to 60 % could be achieved.

With the obtained assessment, the reactions can be further analyzed to identify the main sources of different impact criteria and thereby pinpoint key areas for adaptations to improve the sustainability of the reactions.

**Figure 5:**
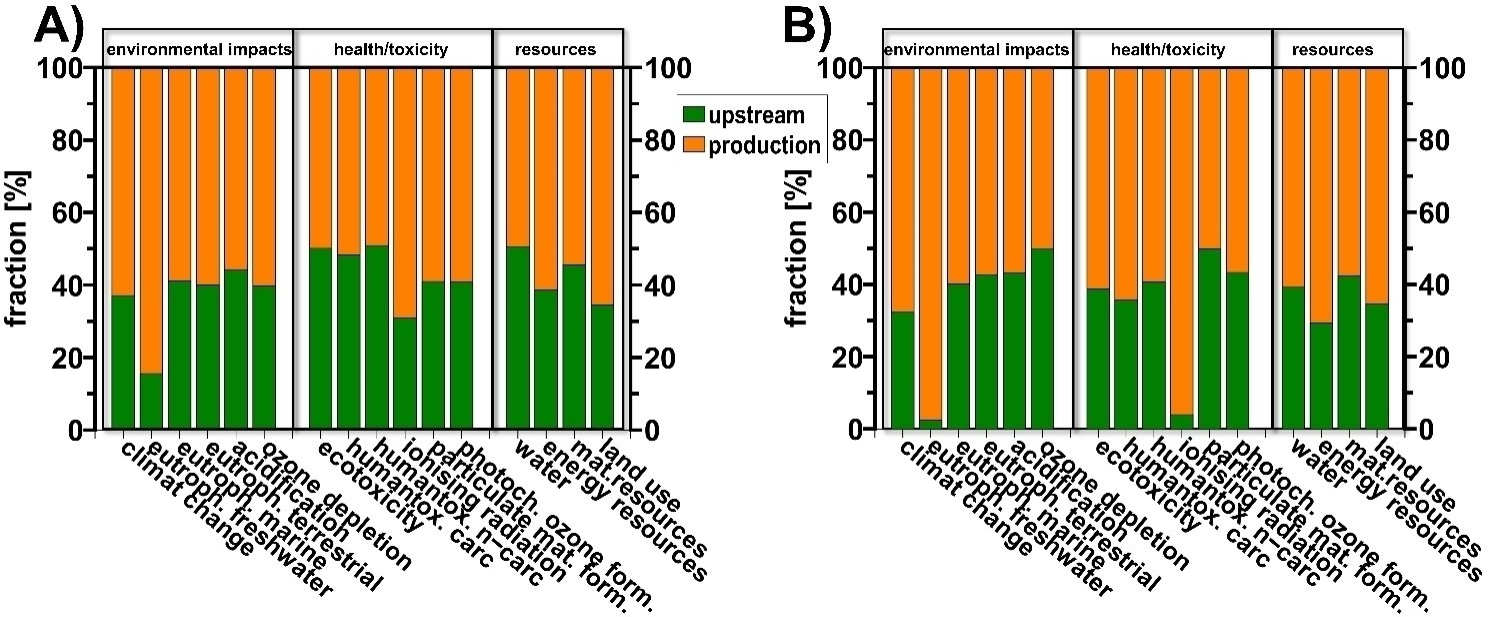
LCA results of PeR (A) and EnzR (B) divided into the impacts of upstream (green) and production (orange).

First, we analyzed the origin of each criterion, whether resulting from upstream processes or the reactions (production in terms of LCA) themselves. The results are shown in Figure 5. It becomes clear that both reactions show a comparable distribution of the impact of the respective criteria between the upstream and the production part. For most criteria upstream part had a share in the range of 40 to 50 %. Thus, the greater impact falls back on the reactions (production) themselves. Exceptions were freshwater eutrophication for both reactions, as well as ionizing radiation additionally for EnzR, whose impact predominantly originates from the production. For ionizing radiation, this is not surprising, as it is associated with electricity generation. With a higher electricity consumption of EnzR, its share also increases. In contrast to the upstream values, which originate from the supply chain and therefore cannot be influenced by us, this applies to the reaction (production) values. With approximately equal values regarding their fractions for upstream or production, both reactions thus have a roughly comparable optimization potential.

**Figure 6:**
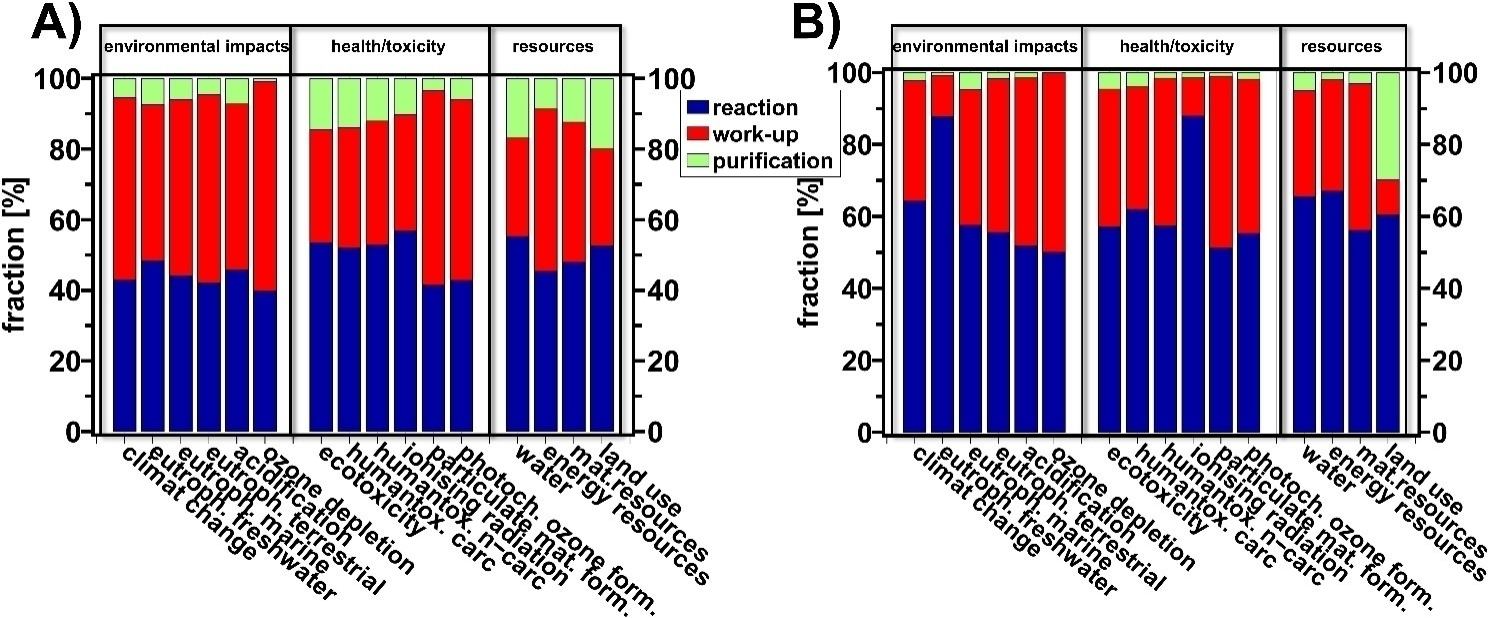
LCA results of PeR (A) and EnzR (B) divided into phases: reaction (blue), work‐up (red) and purification (green).

Next, we analyzed the different phases of the production process (reaction, work‐up including filtration, separation, drying, and evaporation, purification, see Figure 4) regarding their impact on the overall assessment. The share of each step to the total criterion is shown in Figure 6.

For both pathways, we found that the reaction itself contributed the most significant share to the total impact of each criterion. However, this share was slightly higher for EnzR (on average about 60 %), while for PeR, approximately 40 to 50 % was due to the reaction itself. This difference is attributed to the higher energy requirement of EnzR, due to the slightly higher temperature and longer reaction time. The second major contribution is attributed to the work‐up, where the share results less from the chemicals used, but more from the waste generated in this step, which largely stems from the reaction. Here, slightly higher values were observed for PeR, which could be attributed to the waste product of mCPBA (3‐chlorobenzoic acid), or to the lower share of the reaction itself compared to EnzR, consequently increasing the share of the work‐up.

The purification step had a higher share for PeR compared to EnzR. This was expected, as the preparative effort for EnzR was lower due to the non‐necessity of recrystallization, thus saving chemicals and energy.

**Figure 7:**
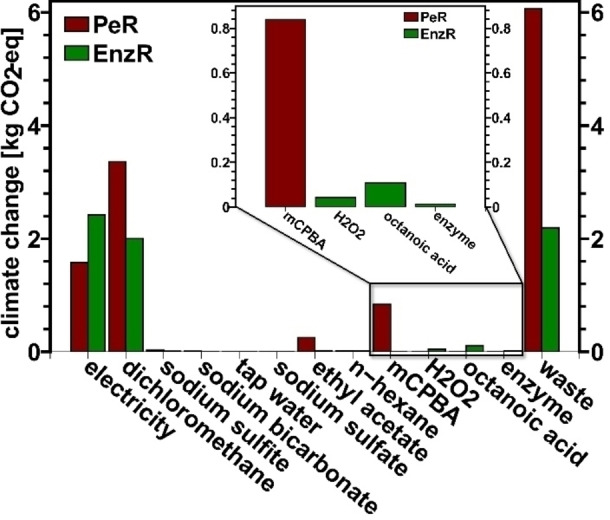
contribution of different factors towards the impact in terms of climate change [kg CO_2_‐Eq] for PeR and EnzR, values are normalized to 10 g dEP (product).

The third aspect we closely examined was the impact of specific chemicals on overall environmental impacts, focusing on the criterion climate change (CO_2_‐emission). We analyzed the effects of electricity demand and different chemicals used in the processes, and the results are presented in Figure 7. The pursuit of greater sustainability through a modified reaction procedure, such as employing enzymatic reactions, includes using more sustainable and renewable chemicals. The results allow for a direct comparison of the environmental impact of chemicals required for the epoxidation. Specifically, mCPBA is replaced by the enzyme, octanoic acid, and hydrogen peroxide.

The cumulative impact of these three components still resulted in lower environmental impacts compared to mCPBA, demonstrating the sustainability of the alternative chemicals. The use of immobilized enzymes, such as CAL‐B in our work, is often associated with greater sustainability, as enzyme separation and potential reuse are easier to achieve. However, our results indicate that even if the enzyme could be reused (disregarding any changes in activity), the overall impact of enzyme recycling would be negligible, contributing only to 0.3 % to the total climate change value for EnzR.

Evaluating the impact of different chemicals facilitated the identification of key contributors to specific environmental criteria. Solvents, electricity, and waste were identified as major factors in CO_2_ production. The highest value was found for the waste from PeR. This significant difference is not only due to the waste product of mCPBA but also to the poorer yield of PeR. Since all values are normalized to a product mass of 10 g, lower yields result in higher values for the respective chemical, as a higher input is needed to obtain the target product mass. This also explains the values for dichloromethane and electricity. Overall, 1.7 times as much dichloromethane is used for EnzR compared to PeR. However, due to the better yield, the overall impact of dichloromethane is lower for EnzR compared to PeR. The same can be observed for electricity, which has an overall value 3.4 times higher for EnzR (see Table S1, supporting information, based on primary data). In short, the higher demand can be explained by the needed heating to 35 °C (instead of room temperature for PeR) and the needed cooling device of the apparatus as this temperature is close to the boiling point of dichloromethane. But again, due to the better yield, this reduces to a much smaller difference in the LCA analysis.

**Figure 8:**
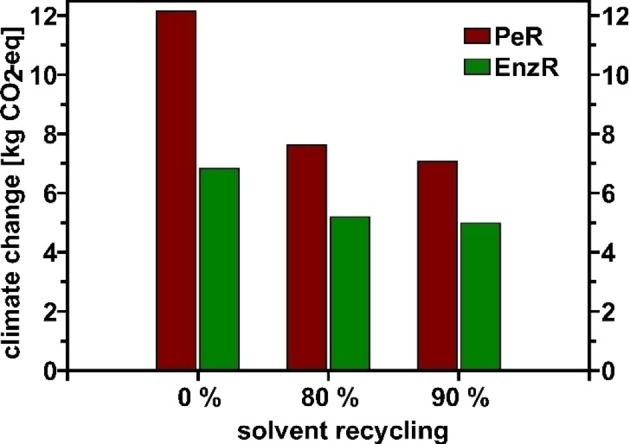
influence of solvent recycling on the total value for climate change for PeR (brown) and EnzR (green).

To reduce the overall impact of waste, recycling of chemicals could be considered. In particular, solvents contribute significantly to the total waste quantities but are also suitable for recycling. As a potential scenario, the changes in CO_2_ emissions under the assumption of different solvent recycling rates were recalculated. The results are shown in Figure 8.

This clearly shows the potential reduction in CO_2_ emissions when solvents are reused. PeR benefits significantly, with CO_2_ emissions decreasing from about 12 kg to 7 kg when the recycling rate is increased to 90 %. The change for EnzR is much smaller. The reason lies again in the significantly better yield of EnzR, which requires less solvent overall compared to PeR.

These considerations also highlight the importance of yields on the overall environmental footprint. In another scenario, we increased the yield of PeR to the same level as EnzR (59.8 %). This might be a realistic scenario, as numerous studies have already reported good to very good yields for epoxidation with mCPBA.[^2^, ^6^, ^7^, ^8^, ^13^] With this increase, the values for the individual criteria decrease accordingly. Recalculated results are shown in Table S2. It now appears that PeR has lower values for the vast majority of criteria, with exceptions in a few ecotoxicity and human toxicity criteria. Under these adjustments, PeR might then be considered the more sustainable reaction, although in the special case of the diepoxidation of EuDI, higher yields might not be accessible due to side reactions, which are under analysis right now.

This clearly shows that the basic evaluation concepts of green chemistry alone are not sufficient to judge the sustainability of a reaction. Precise evaluation using LCA is required. Although our results show sustainability advantages for EnzR under current conditions, the overall result can be strongly influenced by changing conditions (solvent recycling, increase of yield).

## Summary

The sustainability of two reaction routes leading to a bio‐based diepoxy monomer for vitrimers was compared. The focus was set on the epoxidation of alkenes that can be performed either using the petrochemical substance mCPBA or via enzymatic catalysis via CAL‐B. The optimization of both reactions initially showed limitations for the petrochemical routes in terms of yield and epoxide conversion rate. Only a 2.5‐fold stoichiometric excess allowed for nearly complete conversion of the available C‐C double bonds, however with pure yield. The enzymatic route was shown to be a versatile alternative and was optimized with regard to reaction temperature, solvent, co‐substrate, hydrogen peroxide concentrations, and reaction time. A conversion degree of the C‐C double bonds to epoxide moieties that is comparable to the petrochemical reaction was achieved at higher yields.

The assessment of both reactions using green chemistry principles and LCA allowed for a direct comparison with regard to the used chemicals *and* the energy demands on the base of common LCA criteria. The enzymatic routes achieved significant better results for the majority of LCA criteria and thus can be considered the more sustainable of the two reactions. The detailed analysis allows the identification of key optimization points of the total process, including the origin of the used chemicals as well as the reaction work‐up. Key parameters were found in the reaction design (amount of chemicals used, energy needed) and the handling of the resulting waste. Recycling of solvents, for example, led to significant reductions in terms of CO_2_ emissions for both reactions. A scenario analysis with changed yields highlighted their significance. If the problem of low yields of the diepoxidized product in the petrochemical routes could be solved and yields as high as for the enzymatic routes are reached, the petrochemical routes achieved better results in the majority of criteria and thus might then be considered the more sustainable reaction.

## Conclusions

Beside establishing a previously unknown enzymatic synthesis route towards a diepoxy monomer, we showed that applying green chemistry principles alone might not be sufficient to judge about the sustainability of reaction. Using LCA allows for a much more precise evaluation of the reactions, while simultaneously revealing the critical parameters that significantly contribute to the overall environmental footprint. Identifying those is most relevant for potential up‐scaling of reactions. At the same time, we noticed, that a lot of data (*e. g*. enzyme‐specific data) is still missing in the databases used for LCA, when it comes to fine chemicals and we urge the community to work on this issue.

## Conflict of Interests

The authors declare no conflict of interest.

1

## Supporting information

As a service to our authors and readers, this journal provides supporting information supplied by the authors. Such materials are peer reviewed and may be re‐organized for online delivery, but are not copy‐edited or typeset. Technical support issues arising from supporting information (other than missing files) should be addressed to the authors.

Supporting Information

## Data Availability

The data that support the findings of this study are available from the corresponding author upon reasonable request.
