# Supplementary material for: Sustainability of Enzymatic Monomer Synthesis: Evaluation via Comparison of Petrochemical and Enzymatic Alkene Epoxidation by Life Cycle Assessment
Source: ChemSusChem. 2025 Feb 4;18(10):e202402248. doi: 10.1002/cssc.202402248 (PMC12094135; doi:10.1002/cssc.202402248)
Supplement: Supplementary file 1 — Supporting Information [file CSSC-18-e202402248-s001.pdf]

ChemSusChem

Supporting Information

Sustainability of Enzymatic Monomer Synthesis: Evaluation via Comparison of Petrochemical and Enzymatic Alkene Epoxidation by Life Cycle Assessment

Robin Tannert, Sarah Barth, Jakob Hildebrandt, Andreas Taubert, and Jens Weber*

Supporting information

1 Experimental Information

1.1 Materials

Eugenol (Aldrich, 99%), 1,4-dibromobutane (Thermo scientific, 98+%), potassium carbonate (Merck, 99%), potassium iodide (Colok GmbH), 3-chloroperbenzoic acid (Aldrich, ≤77%), sodium sulphite (Merck, 98%), sodium hydrogen carbonate (Aldrich, ≥99,7%), sodium sulphate (Thermo scientific), lipase acrylic resin (Sigma-Aldrich ≥ 5000 U/g) and all solvents (VWR) were used as received without further purification.

1.2 Analysis

The reaction mixture composition was analyzed via UltiMate 3000 HPLC from Thermo SCIENTIFIC with diode array detector-3000 (254 and 280 nm). Analyses was conducted using a EC 150/3 NUCLEOSHELL RP 18plus, 5 µm column, 40 °C, 1 mL/min flow, solvent acetonitrile (A) and water (B), performing the following method: start 20 % (A) until 2 min, to 100 % (A) from 2 to 9 min, 100 % to 20 % (A) from 9 min to 11 min, 20 % (A) until 17 min.

Nuclear magnetic resonance (NMR) spectra were recorded on a BRUKER 400 MHz NMR spectrometer. $CDCl_3$ was used as the solvent. Chemical shifts of ^1H and ^{13}C NMR peaks are reported in ppm against tetramethylsilane as internal standard.

1.3 Detailed synthesis procedure

The molecules synthesized here have already been described and characterized in our previous work.[30] Relevant analytical data and spectra can be obtained from that source. The following synthesis descriptions provide a detailed overview of the exact quantities of chemicals used for the LCA.

Petrochemical reaction (Figure S1):

10 g (26.1 mmol) of EuDI is weighed and dissolved in 100 mL of dichloromethane. Then, 23.2 g (103.5 mmol) of 3-Chloroperbenzoic acid (mCPBA) is added. Any remaining mCPBA is dissolved in an additional 20 mL of dichloromethane and added to the mixture. The flask is placed in a water bath maintained at 20 °C and left for one hour for initial cooling to dissipate reaction heat. Subsequent cooling of the water bath is unnecessary as the heat release diminishes significantly. The mixture is stirred continuously for a total of 12 hours. Afterward, the mixture is filtered to separate precipitated 3-chlorobenzoic acid and washed with 20 mL of dichloromethane.

The organic layer is transferred to a separation funnel using an additional 20 mL of dichloromethane. Here, the mixture is washed three times with 7 mL of saturated sodium sulfite solution followed by three washes with 7 mL of saturated sodium bicarbonate solution. The organic layer is then dried using 1 g of sodium sulfate, filtered, and washed with 10 mL of

dichloromethane. The dichloromethane is evaporated to remove solvent, leaving behind a residue that is recrystallized in n-hexane and ethyl acetate. Complete solubilization requires 5 mL of n-hexane and 21 mL of ethyl acetate. The mixture is cooled to room temperature and filtered. The filtrate is washed with 4.5 mL each of n-hexane and ethyl acetate, yielding 2.42 g of the epoxide product.

Enzymatic reaction (Figure S2Figure S1):

10 g (26.1 mmol) of EuDI is weighed and dissolved in 200 mL of dichloromethane in a three-necked flask equipped with a Liebig condenser. Then, 15.06 mL (100.3 mmol) of octanoic acid is added, and the mixture is heated to 35 °C. Next, 0.8 g of CAL-B enzyme is added, followed by 20 mL of hydrogen peroxide (33%, 218 mmol). The mixture is maintained at these conditions for 24 hours. Afterward, the mixture is filtered to remove the enzyme, and the residue is washed with 20 mL of dichloromethane. The organic layer is transferred to a separation funnel using an additional 20 mL of dichloromethane. In the separation funnel, the mixture is washed three times with 7 mL of saturated sodium sulfite solution and then three times with 7 mL of saturated sodium bicarbonate solution. The organic layer is dried using 1 g of sodium sulfate, filtered, and washed with 10 mL of dichloromethane. The dichloromethane is evaporated, resulting in the precipitation of crystals. The crystals are filtered to yield 5.98 g of the epoxide product.

Optimization procedure of reaction conditions for enzymatic reaction:

1 g Eugenol Diene (0.0026 mol) was dissolved in 20 mL dichloromethane (and water, 1:1). Reaction temperature and amounts of octanoic acid and hydrogen peroxide were added according the parameters as reported under “biotechnological reaction: enzymatic epoxidation” in the main text. For CAL-B 50 mg were added. The mixture was reacted for 20 hours, a small samples withdrawn from the solution, evaporated by dry air and analyzed via HPLC.

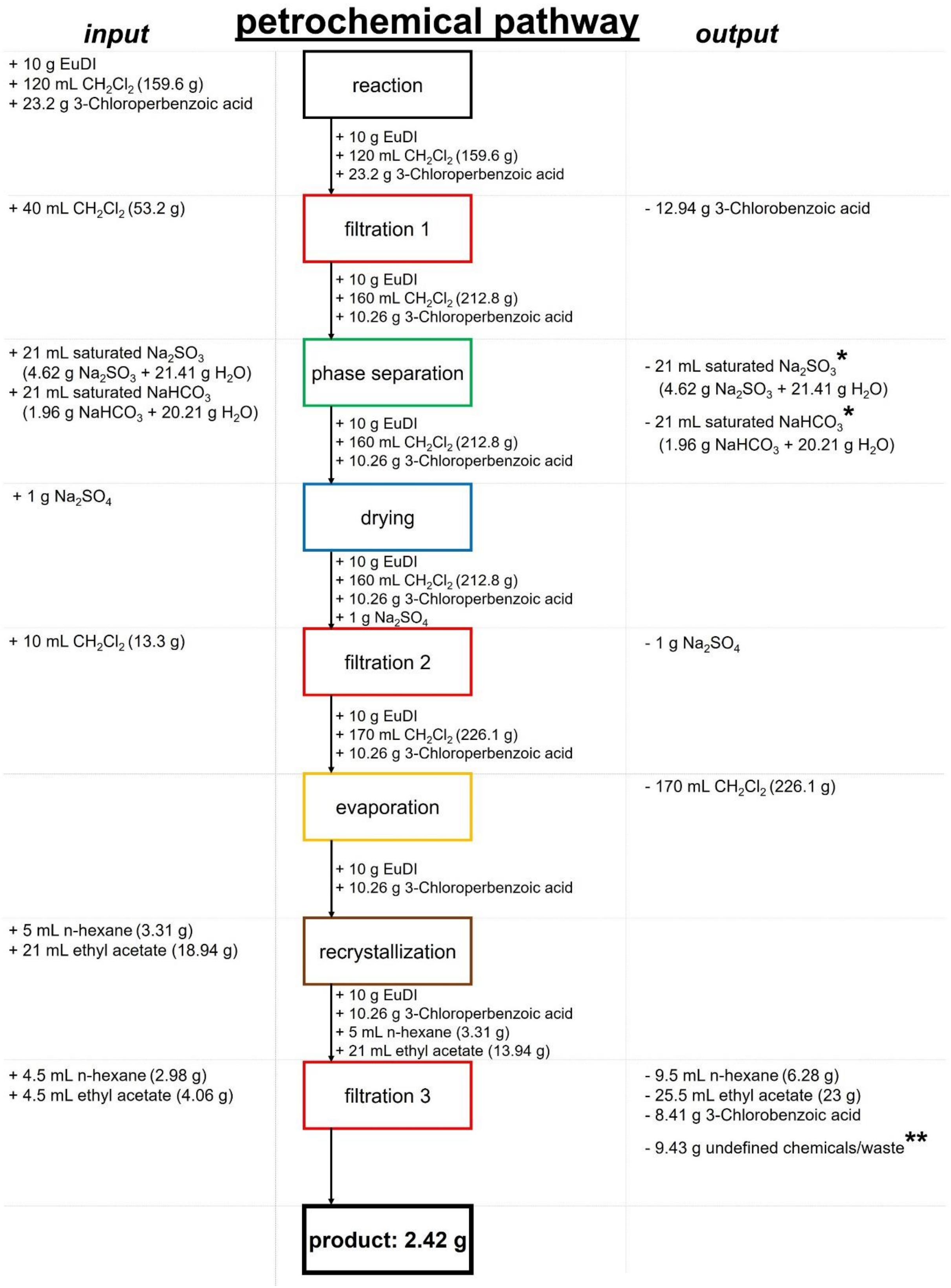

Figure S1 protocol for the synthesis procedure of the epoxidation of EuDI with mCPBA, each procedural step outlines the input and output of chemicals, including the quantities transferred to the subsequent step; *simplification – since the exact concentration of the products(s) from the reaction is unknown, the entire initial chemical input is treated as waste, ** undefined mass to equalize the total massstream

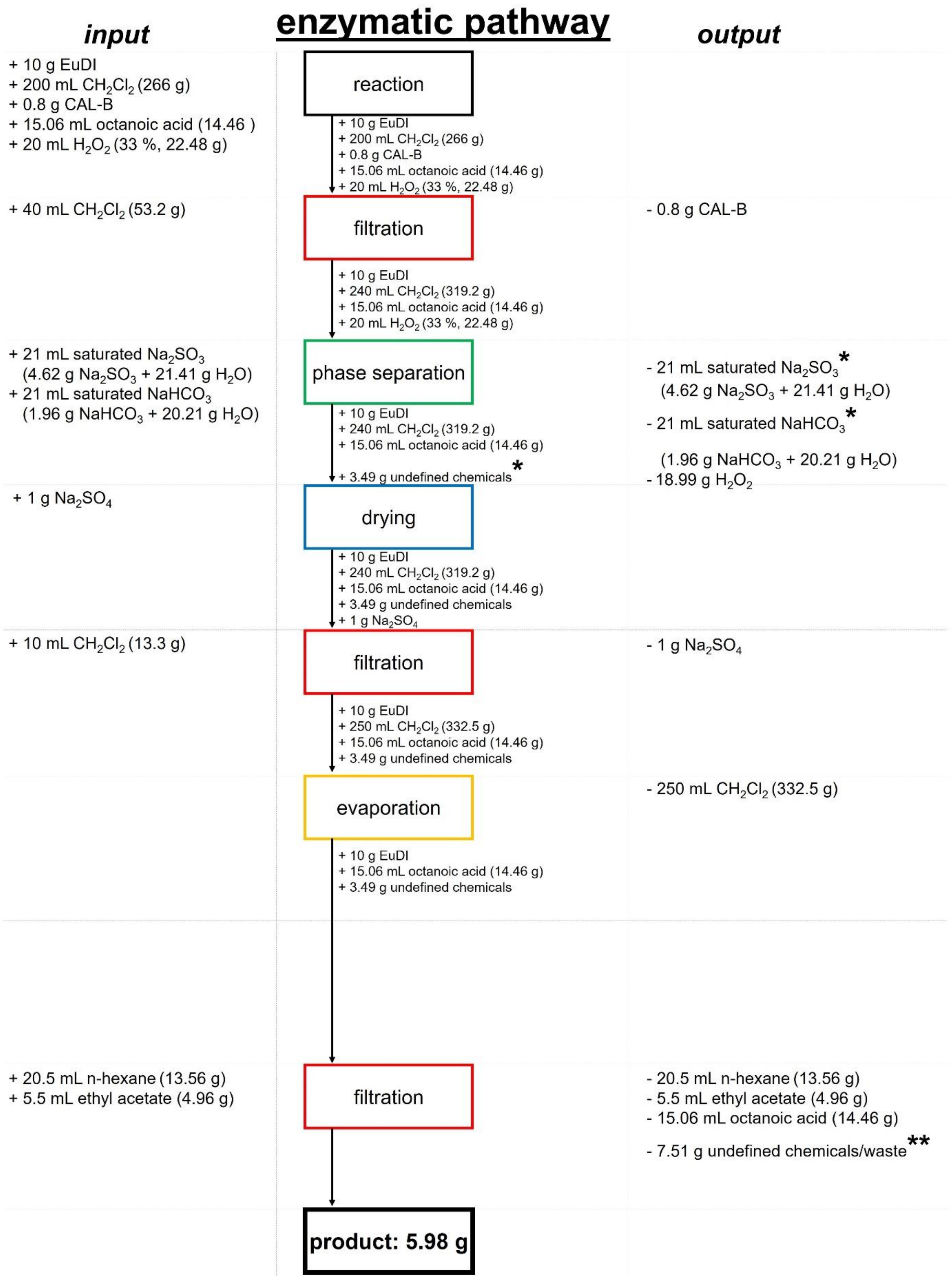

Figure S2 protocol for the synthesis procedure of the epoxidation of EuDI with CAL-B, each procedural step outlines the input and output of chemicals, including the quantities transferred to the subsequent step; * simplification – since the exact concentration of the products(s) from the reaction is unknown, the entire initial chemical input is treated as waste, ** undefined mass to equalize the total mass stream

1.4 Detailed description for determination of power consumption:

The power consumption was recorded using a 4-3680 W energy consumption meter from b1.

Both reactions require power for stirring, heating, and cooling during the first step, the reaction itself. The enzymatic reaction is conducted at an elevated temperature, so the power required to heat the oil bath to the specified temperature was measured. The power consumption for the reaction itself was measured continuously for two hours and then multiplied by the total reaction time. This value includes the power needed for stirring and heating.

Both reactions also require cooling: for the petrochemical reaction, cooling is achieved using a water bath set to 20 °C. For the enzymatic reaction, a condenser is used to avoid solvent evaporation. The condenser is connected (alone) to a cooling circuit operated by a cooling unit from IKA (RC5 basic). The power consumption of the cooling unit (water bath or condenser) was determined over one hour and then multiplied by the total cooling time needed.

During the work-up phase, both reactions involve filtration. The power consumption of the pump used for filtration was measured and multiplied by the filtration time required.

For solvent evaporation, the power consumption to initially heat the water bath to the desired temperature was determined. The power consumption was measured continuously until the target temperature was reached. Subsequently, the power required to maintain this temperature was measured over the entire duration of solvent evaporation. The power consumption to maintain reduced pressure during evaporation was also measured continuously throughout the process.

During the purification step, the power needed to heat the oil bath to the required temperature for separation was measured. The power consumption to maintain this temperature during recrystallization was measured over the entire recrystallization time. The power consumption for filtration during this step was derived from the values determined during the work-up procedure.

Table S1 overview of the determined power consumption for the petrochemical and the enzymatic reaction

	**Petrochemical reaction**	**Enzymatic reaction**
	**Reaction**	
Temperature setting before reaction start	Omitted	12 min, 35 °C<br>power: 0.02 kWh
Reaction	12 h, stirring power: 0.02 kW<br>total (12 h): 0.24 kWh	24 h, 35 °C, power for stirring and heat: 0.04 kW<br>total (24 h): 0.96 kWh
Cooling of the reaction	1 h, cooling unit: 0.055 kW	24 h, cooling unit: 0.055 kW

	total (1 h): 0.055 kWh	total (24 h): 1.32 kWh
**Subtotal reaction**	**0.295 kWh**	**2.3 kWh**
	**Work-up**	
Filtration 1	5 min, power: 0.065 kW<br>total: 0.0054 kWh	5 min, power: 0.065 kW<br>total: 0.0054 kWh
Filtration 2	5 min, power: 0.065 kW<br>total: 0.0054 kWh	5 min, power: 0.065 kW<br>total: 0.0054 kWh
Rotary evaporator temperature setting (50 °C)	4 min, power approx. 1330 W,<br>total: 0.089 kWh	4 min, power approx. 1330 W,<br>total: 0.089 kWh
Rotary evaporator temperature holding (50 °C)	60 min, power 93 W<br>total: 0.093 kWh	60 min, power 93 W<br>Summe: 0.093 kW
Pump pressure 700 mbar	30 min, power: 3 W<br>Summe: 0.0015 kWh	30 min, power: 3 W<br>Summe: 0.0015 kWh
Pump pressure 25 mbar	30 min, power approx. 10 W<br>total: 0.01 kWh	30 min, power approx. 10 W<br>total: 0.01 kWh
Cooling	1 h, cooling unit: 0.055 kW<br>total (1 h): 0.055 kWh	1 h, cooling unit: 0.055 kW<br>total (1 h): 0.055 kWh
**Subtotal**	**0.3763 kWh**	**0.3763 kWh**
	**Purification**	
Temperature setting	Designated temperature (70 °C) after 26 min., power: 0.133 kW<br>total: 0.058 kWh	Omitted
Temperature holding	0.5 h, 70 °C, power for stirring and heat: 0.06 kW<br>total (0.5 h): 0.03 kWh	Omitted
Cooling	0.5 h, cooling unit: 0.055 kW<br>total: 0.0275 kWh	Omitted
Filtration 3	5 min, power: 0.065 kW<br>total: 0.0054 kWh	5 min, power: 0.065 kW<br>total: 0.0054 kWh
**Subtotal**	**0.1209 kWh**	**0.0054 kWh**
**Total**	**0.7922 kWh**	**2.6817 kWh**

2 Scenario analysis with identical yields for both reactions

The results showed that the key factor for the better balance of the enzymatic reaction was the significantly higher yield. Since higher yields with other molecules can be achieved in petrochemical epoxidation, the results were recalculated assuming an identical yield (59.8%).

Table S2 results of different criteria of LCA of petrochemical reaction (PeR) and enzymatic reaction (EnzR) for an identical yield for both reactions (59.8 %, results for PeR were recalculated), divided into three categories: environmental impacts, health/toxicity and resources, higher values (and therewith less sustainable criteria) are highlighted in bold, values are normalized to 10 g dEP

		PeR	EnzR
**Environmental impacts**			
Climate change [kg CO2-eq]		4.912	**6.82**
	Fossil	4.909	**6.61**
	Land use	0.002	**0.17**
	Biogenic	0.005	**0.035**
Eutrophication			
	Freshwater [kg PO4-eq]	0.0031	**0.00379**
	Marine [kg N-eq]	0.00079	**0.009**
	Terrestrial [mol N-eq]	0.0043	**0.084**
Acidification [mol H+-Eq]		0.0225	**0.038**
Ozone depletion [kg CFC-11-Eq]		$2.96*10^{-5}$	**$5.5*10^{-5}$**
**Health/toxicity**			
Ecotoxicity freshwater [CTUe][a]		80.9	**104.2**
	Metals	20.58	**33.54**
	Inorganics	47.29	**68.15**
	Organics	**13.09**	2.52
Human toxicity carcinogenic [CTUh][a]		$1.19*10^{-9}$	**$1.84*10^{-9}$**
	Metals	$8.26*10^{-10}$	**$1.6*10^{-9}$**
	Inorganics	**$2.12*10^{-18}$**	$1.04*10^{-18}$
	Organics	$3.69*10^{-10}$	**$2.46*10^{-10}$**
Human toxicity non-carcinogenic [CTUh][a]		$8.55*10^{-8}$	**$1.13*10^{-7}$**
	Metals	$4.81*10^{-8}$	**$9.62*10^{-8}$**
	Inorganics	$3.63*10^{-8}$	$1.63*10^{-8}$
	Organics	$1.65*10^{-9}$	**$1.75*10^{-9}$**
Ionising radiation [kBq U235-Eq]		0.124	**0.479**
Particulate matter formation [disease incidence]		$3.61*10^{-7}$	**$6.48*10^{-7}$**
Photochemical ozone formation [kg NMVOC-Eq]		0.012	**0.0224**
**Resources**			
Water [m^3 world eq. deprived]		0.779	**1.36**

Energy resources non-renewable [MJ. Net calorific value]	36.23	**80.49**
Material resources metals/minerals [kg Sb-Eq]	$2.1*10^{-5}$	**$3.16*10^{-5}$**
Land use soil quality index	2.88	**15.75**

[a]CTU…comparative toxic unit, index e: environment, index h: human

3 Calculations

Atom economy AE: The atom economy was calculated according to the equation introduced by Anastas and Eghbali.15

$$AE = \frac{molecular\ weight\ product}{molecular\ weight\ reagents}$$

As our product contains both mEP and dEP, which are both seen as products, each fraction is included in the product.

Petrochemical reaction:

$$AE_{PeR} = \frac{414.49 * 0.83 + 398.49 * 0.17}{382.49 + 172.57 * 2} * 100\ \%$$

$$AE_{PeR} = \frac{MW_{dEP} * fraction_{dEP} + MW_{mEP} * fraction_{mEP}}{MW_{EuDI} + MW_{mCPBA} * 2} * 100\ \%$$

$$AE_{PeR} = 56.6\ \%$$

Enzymatic reaction:

$$AE_{EnzR} = \frac{414.49 * 0.8 + 398.49 * 0.2}{382.49 + 144.21 + 34.01} * 100\ \%$$

$$AE_{EnzR} = \frac{MW_{dEP} * fraction_{dEP} + MW_{mEP} * fraction_{mEP}}{MW_{EuDI} + MW_{octanoic\ acid} + MW_{hydrogen\ peroxide}} * 100\ \%$$

$$AE_{PeR} = 73.4\ \%$$

E factor: The E factor was calculated according to the equation of Sheldon.[36]

$$E\ factor = \frac{mass\ waste}{mass\ product}$$

For the mass of waste the accumulated values from Figure S1 and Figure S2 are used.

Petrochemical reaction:

$$E\ factor_{PeR} = \frac{21.35\ g + 5.62\ g + 1.96\ g + \mathbf{226.1\ g} + \mathbf{6.28\ g} + \mathbf{23\ g} + 9.43\ g}{2.42\ g}$$

$$E\ factor_{PeR} = \frac{m_{3-Chlorobenzoic\ acid} + m_{Na_2SO_4} + m_{NaHCO_3} + \mathbf{m_{dichloromethane}} + \mathbf{m_{n-hexane}} + \mathbf{m_{ethylacetate}} + m_{undefined\ waste}}{m_{dEP} + m_{mEP}}$$

$$E\ factor_{PeR} = \ 120.97$$

Enzymatic reaction:

$$E\ factor_{EnzR} = \frac{0.8\ g + 5.62\ g + 1.96\ g + \mathbf{332.5\ g} + \mathbf{13.56\ g} + \mathbf{4.96\ g} + 14.46\ g + 7.51\ g}{5.98\ g}$$

$$E\ factor_{EnzR} = \frac{m_{CAL-B} + m_{Na_2SO_4} + m_{NaHCO_3} + \boldsymbol{m_{dichloromethane}} + \boldsymbol{m_{n-hexane}} + \boldsymbol{m_{ethylacetate}} + m_{undefined\ waste}}{m_{dEP} + m_{mEP}}$$

$$E\ factor_{EnzR} = \ 63.77$$

Both E factors display rather high values because of the laboratory scale, where relativly high amounts of solvents are needed. The E factors are calculated again with the exclusion of all solvents for a better comparison of the impact the remaining waste production of the reactions.

$$E\ factor_{PeR,solvents\ excluded} = \ 15.44$$

$$E\ factor_{EnzR,solvents\ excluded} = \ 5.08$$
